# High-potency multi-strain probiotic formulations for safety and improvement of gastrointestinal function and intestinal health: a randomized controlled clinical trial

**DOI:** 10.3389/fnut.2025.1696243

**Published:** 2025-11-17

**Authors:** Xinru Zhang, Ying Wu, Yiru Jiang, Jiajia Fan, Yao Dong, Shuguang Fang, Jianguo Zhu, Shaobin Gu

**Affiliations:** 1 College of Food and Bioengineering, Henan University of Science and Technology, Luoyang, China; 2 Henan Engineering Research Center of Food Material, Henan University of Science and Technology, Luoyang, China; 3 Wecare Probiotics R&D Centers (WPC), Wecare Probiotics Co., Ltd., Suzhou, China; 4 Henan Engineering Research Center of Food Microbiology, Luoyang, China

**Keywords:** high-potency multi-strain probiotics, safety evaluation, gastrointestinal dysfunction, gut microbiota, multi-strain probiotic formulations

## Abstract

**Background:**

Gastrointestinal dysfunction is a prevalent condition affecting approximately 20–40% of the global population, substantially impairing quality of life. Probiotics have been shown to improve gastrointestinal health by modulating the intestinal microbiota, enhancing epithelial barrier function, and regulating immune responses.

**Methods:**

In this randomized controlled trial, 100 adults (aged 18–65 years) with gastrointestinal dysfunction, diagnosed according to the Citizen’s Intestinal Health and Hygiene Guidelines from the Expert Consensus on Precision Health Communication in China, were enrolled. Participants were randomly assigned to receive either Wec600B (2 sachets/day, 600 billion CFU/sachet, 1,200 billion CFU/day) or Wec1000B (2 sachets/day, 1,000 billion CFU/sachet, 2000 billion CFU/day) for 4 consecutive weeks. Safety outcomes, gastrointestinal symptom improvement rate, immune and inflammatory biomarkers, intestinal barrier function, and gut microbiota diversity were assessed before and after the intervention.

**Results:**

After 4 weeks, both Wec600B and Wec1000B groups demonstrated significant improvement in gastrointestinal symptoms, including indigestion, abdominal pain, reflux, constipation, and diarrhea, without reported adverse events. Levels of fecal calprotectin (FC), neutrophil gelatinase-associated lipocalin (NGAL), and the pro-inflammatory marker FL were reduced, along with intestinal injury indicators such as diamine oxidase (DAO), D-lactic acid (D-LA), and lipopolysaccharide (LPS). In contrast, secretory IgA levels increased. Gut microbiota analysis revealed a significant increase in the relative abundance of beneficial genera, including *Bifidobacterium, Lactobacillus, Blautia,* and *Collinsella*, and a decrease in potentially pathogenic genera such as *Prevotella*, *Escherichia-Shigella*, and *Klebsiella*.

**Conclusion:**

Both Wec600B and Wec1000B high-potency probiotics improved gastrointestinal symptoms and enhanced intestinal health, likely through modulation of gut microbiota composition, reduction of inflammation, and reinforcement of intestinal barrier function.

## Graphical abstract

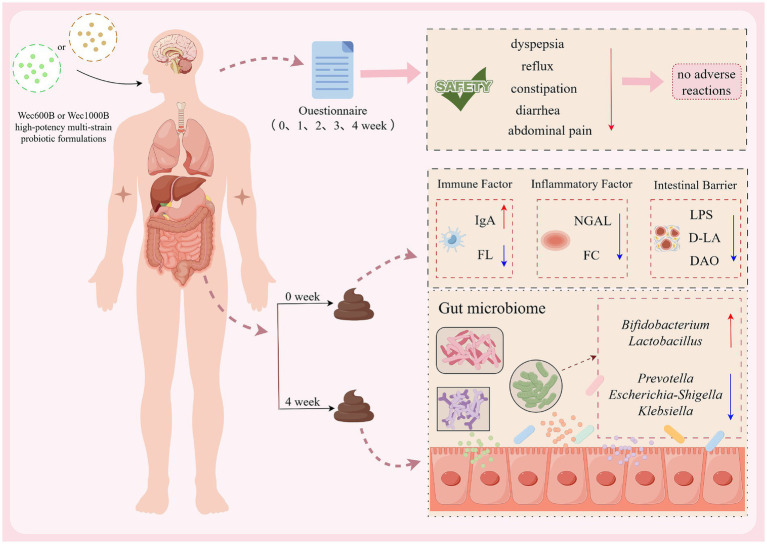

## 1 Introduction

According to the “Epidemiology and Current Status of Diagnosis and Treatment of Functional Gastrointestinal Disorders in China,” the prevalence of functional dyspepsia (FD) (1) among Chinese adults is 23.5%, exceeding the global average of 18%. The prevalence of irritable bowel syndrome (IBS) is 11.2%, with diarrhea-predominant IBS (IBS-D) accounting for 62% of cases. These elevated rates are primarily attributed to high-carbohydrate and high-fat diets (driven by the increasing popularity of delivered meals), psychological stress (a 30% rise in anxiety detection rates), and antibiotic overuse (per capita annual consumption is 1.8 times that of Western countries). Gastrointestinal dysfunction represents a widespread health issue in modern society, characterized by typical symptoms such as indigestion, bloating, constipation, or diarrhea, which significantly impair patients’ quality of life (2).

In addition, with the changes in lifestyle and dietary structure, intestinal flora imbalance has become one of the important factors inducing gastrointestinal dysfunction (3). Probiotics, as active microorganisms that can regulate the balance of intestinal microecology, have shown significant potential in improving gastrointestinal function in recent years (4). They mainly exert beneficial effects through multiple mechanisms, including influencing intestinal barrier function, interacting with intestinal immune cells to alter the local inflammatory environment, and directly or indirectly regulating the existing microbiota (5). Previous studies have demonstrated that probiotics possess both nutritional and immune-regulating benefits, and may help prevent and treat many intestinal diseases related to changes in the intestinal microbiota (6). However, research has found that the causes of gastrointestinal dysfunction are complex, and a single probiotic has limitations and is unable to cover the entire intestine. In contrast, a multi-strain probiotic through the complementary mechanism of ecological niches of different functional characteristics shows significantly better efficacy in alleviating IBS symptoms than any single strain (7). For example, in the case of eradicating *Helicobacter pylori*, the mixture of *Lactobacillus rhamnosus* GG and Bifidobacterium Bb12 is more effective than either single strain given alone (8). Furthermore, existing clinical studies have demonstrated that due to the interaction among the strains, the performance of multi-strain probiotics is superior to that of single-strain probiotics (9, 10). For instance, multi-strain probiotic combinations are more effective in improving gastric emptying and intestinal peristalsis (11), preventing necrotizing enterocolitis (NEC) (12), reducing stress (13), improving the symptoms of adult IBS-D patients (14), and reducing allergic inflammation and improving neurobehavioral outcomes (15).

However, the influence of different combinations of strains and their activities on the efficacy of probiotics still requires further investigation. Currently, it has been found that low-activity probiotic mixtures of different doses can significantly improve gastrointestinal symptoms and regulate the structure of the intestinal flora, especially the higher-activity probiotic mixtures exhibit excellent performance in microbial regulation (16). Nevertheless, systematic research on high-potency multi-strain probiotics remains scarce. Therefore, this study conducts in-depth exploration focusing on high-potency multi-strain probiotics, with an emphasis on their application effects and safety in clinical settings (17). Notably, the clinical efficacy of probiotics is strain-specific and influenced by multiple factors, including strain combinations, viability, dosage, and delivery format (18). High-potency multi-strain probiotic formulations, particularly those produced via freeze-drying technology, are designed to maintain cell viability during storage and gastrointestinal transit, thereby improving colonization potential and functional activity in the gut (19).

Preclinical studies of two proprietary high-potency multi-strain probiotic formulations (Wec600B and Wec1000B; Wecare Probiotics Co., Ltd.) have shown increased microbial diversity and higher short-chain fatty acid production. Nevertheless, there remains a paucity of clinical evidence comparing the efficacy and safety of different strain combinations in improving gastrointestinal function (20). Moreover, despite probiotics generally being recognized as safe (GRAS), potential adverse effects may vary according to strain composition and dosage, underscoring the need for systematic safety evaluation in human trials (20).

This randomized controlled trial assessed the safety and efficacy of Wec600B and Wec1000B in adults with gastrointestinal dysfunction (GID) over 4 weeks. Primary outcomes were changes in gastrointestinal symptoms, inflammatory/immune markers, intestinal barrier measures, and gut microbiota composition; secondary outcomes were adverse events and overall tolerability, to inform evidence-based use of probiotic interventions in GI health.

## 2 Materials and methods

### 2.1 Experimental materials

Two high-potency multi-strain probiotic formulations were evaluated in this study: Wec600B and Wec1000B (Wecare Probiotics Co., Ltd., Suzhou, China). Wec600B comprised the following probiotic strains: *Bifidobacterium animalis* subsp. *lactis* BLa80, *Lacticaseibacillus rhamnosus* LRa05, *Weizmannia coagulans* BC99, *Bifidobacterium longum* subsp. *longum* BL21, *Lactobacillus acidophilus* LA85, *Lacticaseibacillus paracasei* LC86, *Bifidobacterium breve* BBr60, *Pediococcus acidilactici* PA53, and *Lactiplantibacillus plantarum* Lp05, with dextrin as the carrier. Each 3.0 g sachet contained a total viable count of 600 billion colony-forming units (CFU). Wec1000B contained the same probiotic strains and dextrin in identical proportions, with each 3.0 g sachet providing 1,000 billion CFU. Participants in both groups consumed two sachets per day for the 4-week intervention period.

### 2.2 Study design

**Figure 1:**
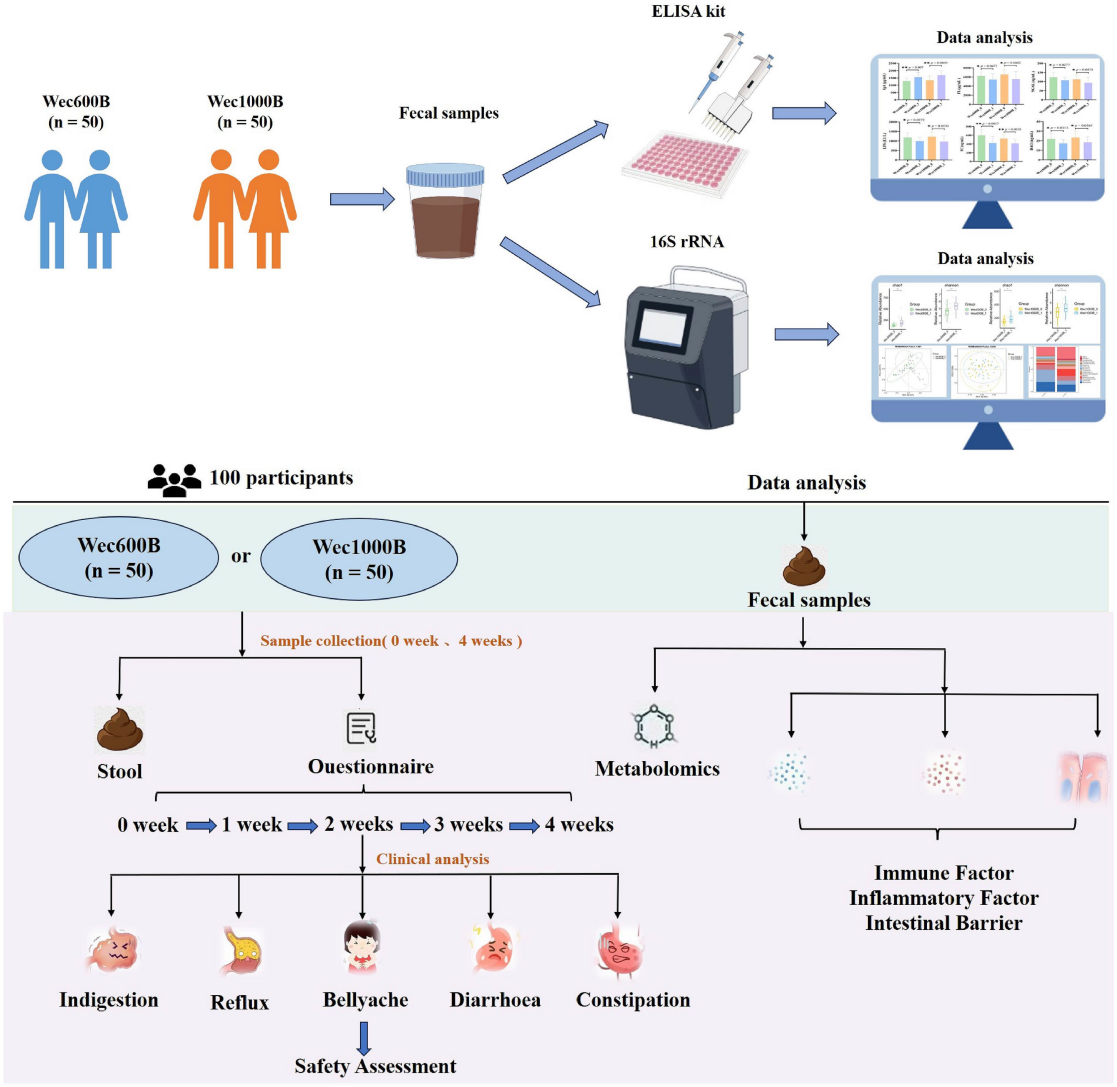
Research design diagram.

This randomized, double-blind, parallel-group clinical trial enrolled adults with abnormal gastrointestinal function, without gender restrictions. As shown in Figure 1, a total of 100 eligible participants from Luoyang, Henan Province were recruited on a voluntary basis and randomly assigned, in a 1:1 ratio, to either the Wec600B group or the Wec1000B group (*n* = 50 per group). Baseline demographic characteristics, including age, gender and cultural background, did not differ significantly between the two groups. Participants randomized to Wec600B were administered two sachets daily (3.0 g/sachet; 600 billion CFU per sachet), and those randomized to Wec1000B received two sachets daily (3.0 g/sachet; 1,000 billion CFU per sachet) for 4 weeks. The formulation was administered after lunch and dinner respectively, and was reconstituted only with warm water (approximately 37 °C) for consumption—this temperature was controlled to avoid damaging the probiotic strains. No other food or beverages were allowed to be consumed concurrently with the product during intake. During the intervention period, participants are required to adopt a healthy dietary pattern with a reasonable dietary structure characterized by “diversity of foods and balanced proportions,” with reference to *Dietary Guidelines for Chinese Residents (2022)*. This can be briefly summarized by the “Plate Method”: staple foods (including grains and tubers) accounting for approximately 1/3 of the plate, vegetables and fruits accounting for about 1/2 of the plate, high-quality protein accounting for roughly 1/6 of the plate, and oils, salt and sugar to be consumed in “trace amounts.” The consumption of fermented foods and supplements is not permitted. Regarding physical activity levels, no additional control was required in this study. Participants were only asked to ensure that their physical activity levels throughout the study period (from the start to the end of the study) remained consistent with their activity levels prior to the study (“pre-intervention”).

Follow-up visits were conducted at weeks 1, 2, 3, and 4 to monitor compliance and record any adverse events, including gastrointestinal discomfort and other complications. Fecal samples were collected at the Affiliated Hospital of Henan University of Science and Technology at baseline (week 0) and post-intervention (week 4). Compliance was assessed by reviewing participant diary cards and collecting all unused products and empty sachet packaging at the end of the study. This trial was registered with ClinicalTrials.gov, number NCT06781814.

### 2.3 Inclusion and exclusion criteria

Inclusion Criteria: Voluntary participation, written and signed informed consent, agreement to participate in this study; ability to complete the study as per the protocol requirements; age between 18 and 65 years; meeting the diagnostic criteria for gastrointestinal dysfunction as stipulated in the “Citizen’s Guide to Intestinal Health and Hygiene” of the “Expert Consensus on Precision Health Communication in China”; symptoms: irregular bowel movements, loose or hard stools, intestinal rumbling in the abdomen, abdominal distension, belching, hiccups, excessive intestinal gas, abdominal pain, acid reflux, heartburn, pain in the stomach or abdomen when hungry, nausea; signs: abdominal pain and distension, diarrhea and constipation, indigestion, acid reflux, bad breath and flatulence, skin problems, changes in stool color and shape.

Exclusion Criteria: Use of drugs that affect the intestinal flora (including antibiotics, probiotics, intestinal mucosal protectants, traditional Chinese patent medicines, etc.) for more than one week continuously within one month before screening; short-term use of substances with similar functions to the study, affecting the judgment of results; use of antibiotics during illness; participants with liver and kidney insufficiency, uncontrolled autoimmune diseases, infectious diseases, cardiovascular diseases, neurological disorders, or malignant tumors; those allergic to the components of the multi-strain probiotic formulations used in this study; pregnant or lactating women or those with recent plans for pregnancy; participants who cannot participate in the study due to personal reasons; other participants deemed unsuitable for participation by the researchers.

### 2.4 Sample size and randomization

Two types of control designs were adopted: self-control and inter-group control. The participants were randomly divided into the Wec600B group or the Wec1000B group, considering the main factors that might affect the results such as age, gender, and dietary factors, and conducting an equilibrium test to ensure the comparability between the groups.

The trial was powered for a two-sided comparison of two independent proportions, with the primary endpoint defined as the proportion of participants achieving a prespecified improvement in gastrointestinal dysfunction at the primary assessment visit. Based on prior studies and our pilot data, we assumed an absolute between-group difference of 25 percentage points. Using R (pwr package; pwr.2p.test) with *α* = 0.05 and 80% power, the required sample size was 45 participants per group. Allowing for 10% anticipated attrition, the target enrollment was increased to 50 per group (total *N* = 100). Participants were randomized 1:1 to the two probiotic groups using computer-generated permuted blocks (variable block sizes), without stratification, with allocation concealment maintained via a centralized randomization list. The randomization seed and algorithm were recorded and archived prior to enrollment. The intention-to-treat (ITT) population was specified as primary; no per-protocol or other sensitivity analyses were prespecified.

### 2.5 Primary and secondary observation indicators

The main observation indicator is to assess the safety of the two high-potency multi-strain probiotic formulations. Based on the weekly follow-up records of taking the high-potency multi-strain probiotic formulations, the evaluation indicators include the daily defecation frequency within a week, whether the stool is loose or hard, whether there is abdominal distension or acid reflux, etc., to evaluate the safety of gastrointestinal symptom improvement. The secondary observation indicators are to evaluate the effects on immune factors, inflammatory factors, intestinal barrier, and intestinal flora based on the fecal sample test results at weeks 0 and 4 after taking the high-potency multi-strain probiotic formulations.

### 2.6 Detection methods

Fecal routine examination is used to evaluate the fecal characteristics; the “Questionnaire Star” platform (21, 22) —China’s largest professional survey platform, certified to ISO 26362—is used to conduct a questionnaire survey for the research participants, mainly investigating the basic information of the research participants, collecting symptom scores for abdominal rumbling, abdominal distension, hiccups, intestinal flatulence, abdominal pain, heartburn, hunger pain, nausea, etc. to evaluate the safety indicators; ELISA kit method is used to measure IgA, FL, NGAL, FC, LPS, DAO, D-LA in feces, and the measurement process strictly follows the operating methods in the manual; 16S rRNA gene sequencing technology is used to analyze the intestinal flora.

### 2.7 Statistical methods

Data are analyzed and processed using Graphpad Prism 10.0 and SPSS 22.0 statistical software. Measurement data are expressed as ( $\bar{x}\pm s$ ), and t-test is used. *p* < 0.05 is considered statistically significant.

## 3 Results

### 3.1 Baseline characteristics

A total of 100 participants from Luoyang, Henan, completed a 4-week intervention study. In the Wec600B product group, there were 23 males and 27 females, with an average age of 26.12 ± 11.3 years. In the Wec1000B product group, there were 22 males and 28 females, with an average age of 30.08 ± 12.98 years. The participants in both groups had similar age, gender, and cultural background levels, and there was no statistically significant difference between the two groups (*p* > 0.05), making them comparable. As shown in Table 1.

**Table 1:** Baseline characteristics of the two groups of study participants.

Product	Gender [Proportion (%)]		Age (Years, $\bar{x}\pm s$ )	BMI		
	Male	Female		Before	After	*p*-value
Wec600B(*n* = 50)	23 (46%)	27 (54%)	26.12 ± 11.30	23.56 ± 2.62	23.37 ± 2.47	0.7667
Wec1000B(*n* = 50)	22 (44%)	28 (56%)	30.08 ± 12.98	23.47 ± 2.78	23.21 ± 2.59	0.6894

### 3.2 Safety evaluation

#### 3.2.1 Effects of Wec600B and Wec1000B interventions on gastrointestinal function symptoms

**Figure 2:**
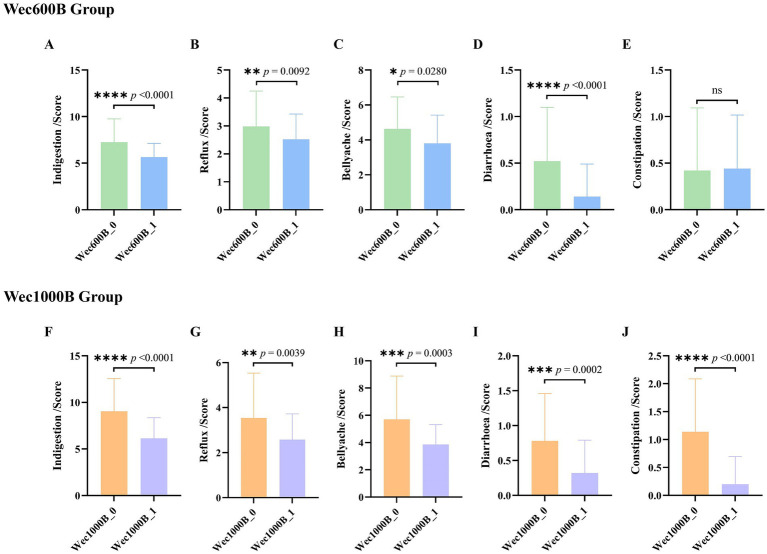
The effects of Wec600B and Wec1000B on gastrointestinal functional symptoms before and after intervention. **(A,F)** The dyspepsia scores of the Wec600B group and the Wec1000B group. **(B,G)** The reflux scores of the Wec600B group and the Wec1000B group. **(C,H)** The abdominal pain scores of the Wec600B group and the Wec1000B group. **(D,I)** The diarrhea scores of the Wec600B group and the Wec1000B group. **(E,J)** The constipation scores of the Wec600B group and the Wec1000B group. Wec600B_0 and Wec1000B_0 represent the pre-intervention status of the Wec600B group and the Wec1000B group; Wec600B_1 and Wec1000B_1 represent the post-intervention status of the Wec600B group and the Wec1000B group.

To assess the safety of the high-potency multi-strain probiotic formulations for the participants in the study, the impact on gastrointestinal discomfort symptoms was observed, and relevant safety evaluations were conducted. In this study, symptom scoring questionnaires for heartburn, acid reflux, hunger-related epigastric pain, nausea, belching and flatulence, borborygmi, abdominal bloating, increased flatulence, abdominal pain, constipation, and diarrhoea were collected at weeks 0, 1, 2, 3, and 4, according to the GSRS scoring scale, which was divided into 5 dimensions (23): dyspepsia (abdominal bloating, increased flatulence, belching and borborygmi), abdominal pain (abdominal pain, hunger-related epigastric pain, nausea), reflux (heartburn and acid reflux), constipation, and diarrhoea. Each item was rated from no symptoms to very severe based on severity, with a total of 7 levels (24). Among them, the 4 items included in dyspepsia were rated from no symptoms to very severe based on severity, with a total of 6 levels; constipation and diarrhoea were rated from no discomfort to severe discomfort, with a total of 4 levels (25). The results are shown in Tables 2, 3 and Figure 2. At one week of intervention, symptoms such as dyspepsia and reflux were significantly relieved, and other symptoms also improved. The improvement of intestinal function gradually strengthened with the extension of intervention time, and participants experienced reduced gastrointestinal discomfort. Both constipation and diarrhoea symptoms were improved. Mild loose stools significantly decreased after one week of intervention and continued to improve in the subsequent period; moderate and severe loose stools were also rapidly relieved after intervention and were almost absent in the fourth week. In summary, after four weeks of high-potency multi-strain probiotic intervention, the gastrointestinal symptoms of the study participants were significantly reduced compared to the baseline level, and constipation and diarrhoea could be effectively reduced. There were no adverse reactions. It can be observed that the symptom improvement effect of the Wec1000B group is better than that of the Wec600B group, indicating that the high-potency multi-strain probiotic preparation has better safety and gastrointestinal regulatory effects, and is more conducive to improving gastrointestinal health.

**Table 2:** Analysis of gastrointestinal function questionnaire for Wec600B product.

Domain	Evaluation	Score	*p*-value (vs. Baseline)	95% CI (vs. Baseline)
Indigestion	Baseline	7.24 ± 2.52	-	-
	W1	6.36 ± 2.25	0.050	0.27880, 1.48120
	W2	6.62 ± 2.07	0.084	−0.08529, 1.32529
	W3	6.64 ± 1.78	0.126	−0.17459, 1.37459
	W4	5.64 ± 1.48	<0.001	0.92551, 2.27449
Reflux	Baseline	2.98 ± 1.27	-	-
	W1	2.46 ± 0.91	<0.001	0.22588, 0.81412
	W2	2.38 ± 0.73	<0.001	0.30723, 0.89277
	W3	2.50 ± 0.86	0.004	0.15908, 0.80092
	W4	2.52 ± 0.91	0.009	0.11930, 0.80070
Bellyache	Baseline	4.62 ± 1.84	-	-
	W1	3.96 ± 1.26	0.010	0.16405, 1.15595
	W2	3.82 ± 1.10	0.004	0.26446, 1.33554
	W3	3.92 ± 1.56	0.040	0.03226, 1.36774
	W4	3.80 ± 1.62	0.028	0.09217, 1.54783
Diarrhoea	Baseline	0.52 ± 0.58	-	-
	W1	0.52 ± 0.65	1.000	−0.20702, 0.20702
	W2	0.46 ± 0.58	0.583	−0.15796, 0.27796
	W3	0.28 ± 0.54	0.027	0.02857, 0.45143
	W4	0.14 ± 0.35	<0.001	−0.20881, 0.55119
Constipation	Baseline	0.42 ± 0.67	-	-
	W1	0.28 ± 0.57	0.070	0.01202, 0.29202
	W2	0.38 ± 0.60	0.687	0.15856, 0.23856
	W3	0.34 ± 0.56	0.322	0.08077, 0.24077
	W4	0.44 ± 0.58	0.837	0.21462, 0.17462

**Table 3:** Analysis of gastrointestinal function questionnaire for Wec1000B product.

Domain	Evaluation	Score	*p*-value (vs. Baseline)	95% CI (vs. Baseline)
Indigestion	Baseline	9.04 ± 3.52	-	-
	W1	7.22 ± 2.38	0.003	0.62763, 3.01237
	W2	6.80 ± 2.03	0.000	1.10042, 3.37958
	W3	6.30 ± 1.97	0.000	1.60856, 3.87144
	W4	6.14 ± 2.21	0.000	1.73391, 4.06609
Reflux	Baseline	3.54 ± 1.99	-	-
	W1	2.66 ± 1.54	0.008	0.23398, 1.52602
	W2	2.76 ± 1.17	0.019	0.13162, 1.42838
	W3	2.56 ± 0.95	0.002	0.36053, 1.59947
	W4	2.58 ± 1.14	0.004	0.31527, 1.60473
Bellyache	Baseline	5.70 ± 3.18	-	-
	W1	4.84 ± 2.16	0.117	−0.21968, 1.93968
	W2	4.32 ± 1.52	0.007	0.39030, 2.36970
	W3	4.02 ± 1.45	0.001	0.69837, 2.66163
	W4	3.86 ± 1.46	0.000	0.8572, 2.82248
Diarrhoea	Baseline	0.78 ± 0.68	-	-
	W1	0.52 ± 0.65	0.053	−0.00309, 0.52309
	W2	0.54 ± 0.71	0.086	−0.03486, 0.51486
	W3	0.42 ± 0.61	0.006	0.10404, 0.61596
	W4	0.32 ± 0.47	0.000	0.22809, 0.69191
Constipation	Baseline	1.14 ± 0.95	-	-
	W1	0.50 ± 0.74	0.000	0.30332, 0.97668
	W2	0.50 ± 0.74	0.000	0.30332, 0.97668
	W3	0.48 ± 0.71	0.000	0.32818, 0.99182
	W4	0.20 ± 0.49	0.000	0.63992, 1.24008

#### 3.2.2 Adverse reactions

During the trial, by observing the participants’ intake of Wec600B and Wec1000B high-potency multi-strain probiotic formulations weekly, no adverse reactions were observed in both groups, indicating good safety.

### 3.3 Effects of Wec600B and Wec1000B interventions on immune, inflammatory, and gut barrier markers

**Figure 3:**
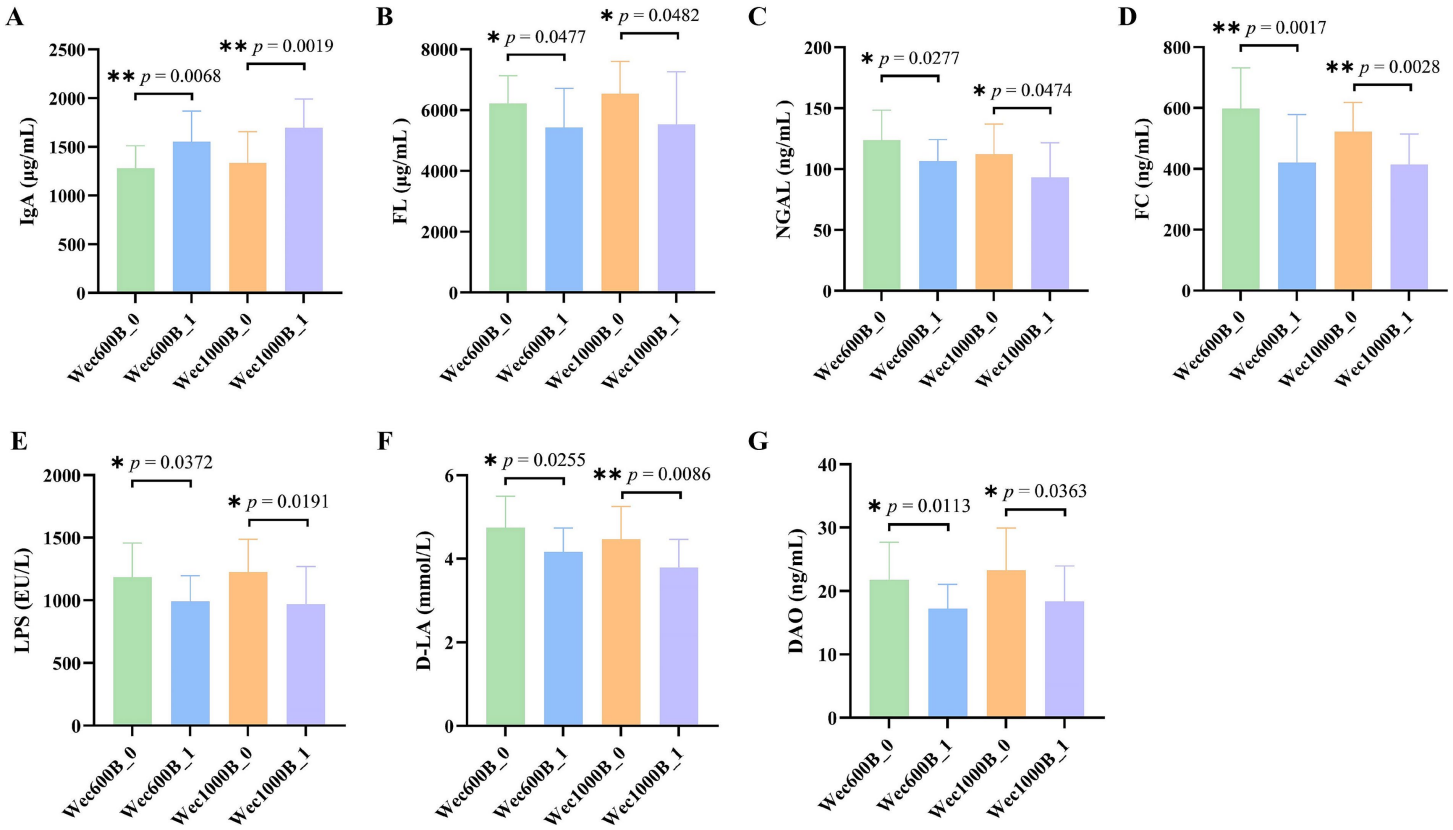
Effects of Wec600B and Wec1000B interventions on immune, inflammatory, and gut barrier markers in feces before and after intervention. **(A)** IgA. **(B)** FL. **(C)** NGAL. **(D)** FC. **(E)** LPS. **(F)** D-LA. **(G)** DAO. Wec600B_0 and Wec1000B_0 represent the pre-intervention status of the Wec600B group and the Wec1000B group; Wec600B_1 and Wec1000B_1 represent the post-intervention status of the Wec600B group and the Wec1000B group.

The intestine is not only an important part of the digestive system, but also the largest immune organ in the human body. 70% of the body’s immunity originates from the intestine. There is a close interrelationship between the intestinal microbiota and the immune system. The intestinal microbiota and its metabolites regulate the immune system through various pathways and play an important role in maintaining the host’s immune homeostasis. Immunoglobulin (IgA) is the most common type of antibody in the human immune system. It is secreted by specialized cells in the mucosa. IgA can inhibit microbial attachment to the respiratory epithelium, slow down virus reproduction, and play an important immune barrier role. It has antibody activity against certain viruses, bacteria, and general antigens and is the first line of defense against pathogen invasion (26, 27). Fecal lactoferrin (FL) is a sugar protein that binds to iron and has immune function. It is an important component of secondary granules of neutrophils and its content increases in inflammatory diseases. When the intestine is inflamed, neutrophils infiltrate the intestinal mucosa, causing an increase in lactoferrin (28). This study measured the levels of IgA and FL in the feces of the study participants before and after the intervention. The results are shown in Figures 3A,B. The data indicate that there was no difference in the baseline between the two groups before the intervention (*p* > 0.05), after 4 weeks of intervention, the IgA levels in both groups significantly increased (*p* < 0.01), with a 21.4189% increase in the Wec600B group and a 26.8608% increase in the Wec1000B group. The FL levels in both groups significantly decreased (*p* < 0.05), with a 12.647% decrease in the Wec600B group and a 15.442% decrease in the Wec1000B group. It is indicated that the high-potency multi-strain probiotics may regulate the intestinal flora, inhibit the growth of pathogenic bacteria, effectively adjust the levels of immune factors in the body, enhance the immune defense ability, and it can be observed that the high-potency multi-strain probiotics have a more significant effect in immune regulation compared to the other group.

Persistent gastrointestinal problems are a type of chronic inflammation. This inflammatory state often does not cause obvious clinical symptoms, but persists for a long time, with continuously elevated levels of inflammatory mediators in the body. Over the long term, it will affect human health. Studies have found that probiotics can regulate the inflammatory response in the body and reduce the damage caused by chronic inflammation to the body. Calprotectin (FC) is a cytoplasmic protein complex that is constitutively expressed in neutrophils and is released when migrating to the intestinal mucosa during intestinal inflammation. In chronic inflammatory diseases, calprotectin can promote the disease process through binding to cytokine receptors and the production of reactive oxygen species (29). Neutrophil gelatinase-associated lipocalin (NGAL) is a lipid carrier protein that is lowly expressed in neutrophils, kidneys, colon, and lung epithelial cells. It is a marker of kidney structural damage, systemic inflammation, and oxidative stress. NGAL can also participate in the glycolipid metabolism and chronic inflammatory response of cells (30, 31). This study measured the levels of FC and NGAL in feces before and after intervention. The results are shown in Figures 3C,D. compared with the baseline, the NGAL level in the probiotic groups decreased significantly after intervention (*p* < 0.05): the Wec600B group showed a 13.689% reduction, while the Wec1000B group exhibited a 16.848% reduction. In terms of the magnitude of reduction, the Wec1000B group demonstrated superior efficacy in lowering NGAL levels and alleviating inflammation compared with the Wec600B group; the FC level significantly decreased (*p* < 0.01), with a reduction of 29.665% in the Wec600B group and 20.574% in the Wec1000B group. This indicates that probiotic intervention may reduce intestinal damage and lower the levels of NGAL and FC by regulating the intestinal flora, inhibiting the growth of pathogenic bacteria, and modulating the intestinal immune response. It suggests that high-potency multi-strain probiotics can effectively reduce intestinal inflammation and promote intestinal health homeostasis in the body.

The early pathogenic processes of many complex diseases often start with damage to the gastrointestinal (GI) barrier function (32, 33). When various factors damage the intestinal barrier function, it can lead to an increase in intestinal permeability, resulting in a series of pathological changes such as the translocation of intestinal flora (34). Lipopolysaccharide (LPS), also known as endotoxin, mainly translocates across the cell membrane through the transcellular pathway. When the intestinal barrier function is impaired, intestinal permeability increases, and bacterial endotoxins (such as LPS) can be transferred to the circulatory system, increasing the level of LPS in the serum of the organism. Therefore, the level of LPS is also considered a potential marker for increased intestinal permeability (35). D-lactic acid (D-LA) is a fermentation product of intestinal resident bacteria and has a very low level in healthy individuals. However, in the case of loss of intestinal barrier function, the increase in the paracellular pathway leads to an increase in intestinal permeability, and a large amount of D-LA enters the bloodstream through the damaged mucosa, causing an increase in the level of D-LA in the blood (36). The detection of D-LA levels in feces can also, to a certain extent, assess intestinal barrier damage (37). Studies have confirmed the relationship between fecal DL-lactic acid and intestinal inflammation or malabsorption (38). Diamine oxidase (DAO) is an intracellular enzyme located on the intestinal mucosa and has high potency. Its biological activity is affected when intestinal mucosal cells are damaged, and it is an important indicator for judging the function of the intestinal mucosal barrier (39). This study measured the levels of LPS, D-LA, and DAO in feces before and after intervention. The results are shown in Figures 3E–G, after the intervention, the levels of LPS, D-LA, and DAO were significantly reduced (*p* < 0.05). In the Wec600B group, they decreased by 16.327, 13.7308, and 20.9419% respectively; in the Wec1000B group, they decreased by 20.916, 15.199, and 21.1466%, respectively., indicating that high-potency multi-strain probiotics may effectively improve intestinal health by regulating the intestinal flora, enhancing intestinal barrier function, and regulating immune responses. The results of the two groups of interventions showed that the intervention effect of the Wec1000B group was better than that of the Wec600B group. This indicates that the higher-potency multi-strain probiotic preparations play a significant role in maintaining intestinal homeostasis and enhancing the body’s resistance.

### 3.4 Regulation of gut microbiota

#### 3.4.1 Changes in bacterial diversity and richness

**Figure 4:**
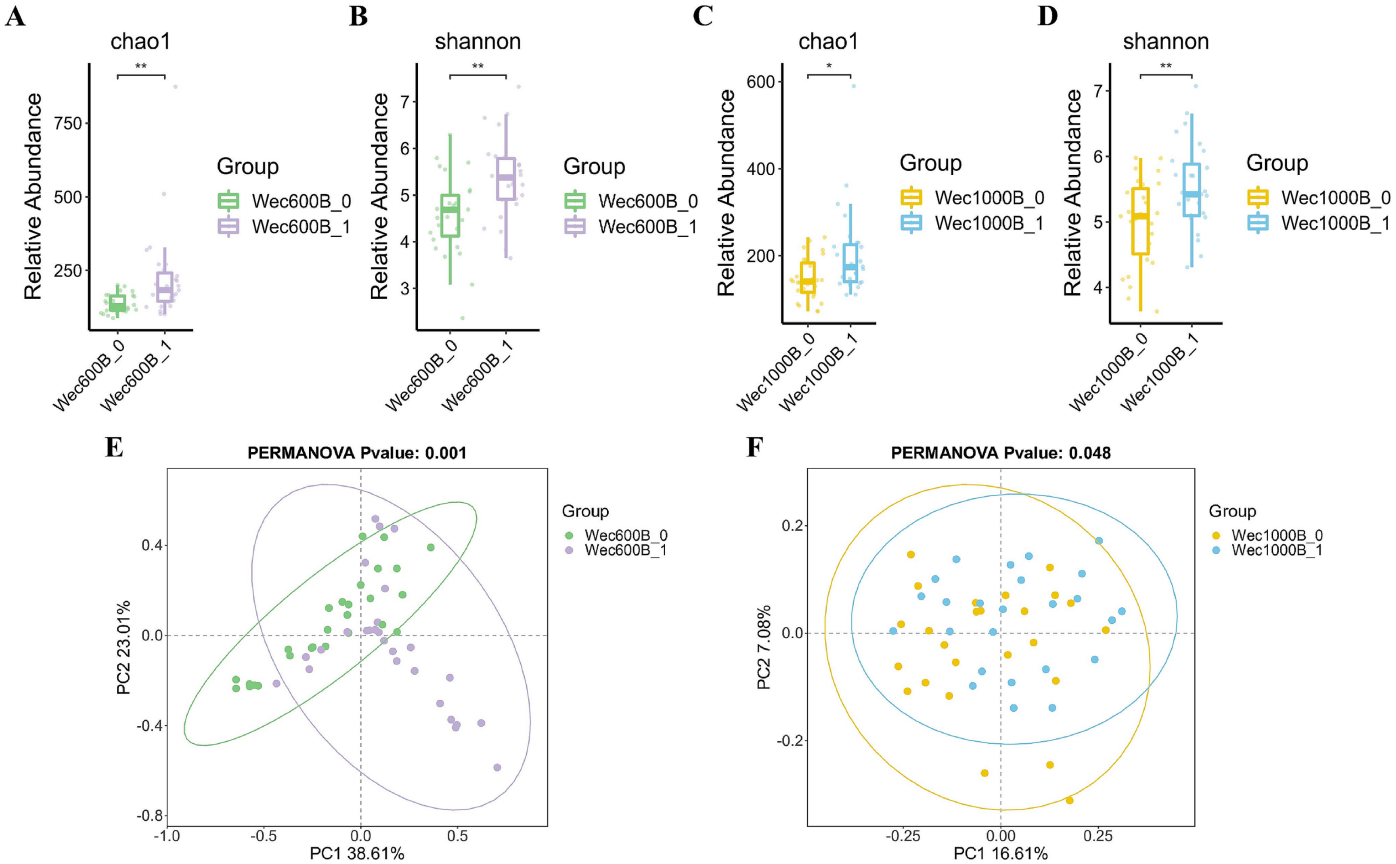
The effects of Wec600B and Wec1000B interventions on the changes in bacterial community diversity and richness in feces. **(A)** Chao1 plot of the Wec600B group. **(B)** Shannon plot of the Wec600B group. **(C)** Chao1 plot of the Wec1000B group. **(D)** Shannon plot of the Wec1000B group. **(E)** PCoA plot of the Wec600B group. **(F)** PCoA plot of the Wec1000B group. Wec600B_0 and Wec1000B_0 represent the pre-intervention status of the Wec600B group and the Wec1000B group; Wec600B_1 and Wec1000B_1 represent the post-intervention status of the Wec600B group and the Wec1000B group. *p < 0.05; **p < 0.01; ***p < 0.001.

In order to investigate the degree of Alpha diversity and Beta diversity of species within the biological intestinal microbiota before and after the intervention with Wec600B and Wec1000B high-potency multi-strain probiotic formulations, the changes in the microbiota were evaluated by analyzing the Chao1, Shannon index, and principal coordinate analysis (PCoA). As shown in Figures 4A–D, the results indicated that after the intervention with Wec600B and Wec1000B high-potency multi-strain probiotic formulations, the Alpha diversity of the microbial community changed, and these indices increased in both groups, showing significant differences. Beta diversity is an indicator used to analyze the differences in the composition and structure of microbial communities between different samples or groups. Figures 4E,F evaluated the effect of Wec600B and Wec1000B composite probiotic formulations on the Beta diversity of the intestinal microbiota through principal coordinate analysis (PCoA). The results showed that the microbial community structure in the Wec600B group before intervention (Wec600B_0) and after intervention (Wec600B_1) presented a highly significant separation in PCoA (PERMANOVA *p* value = 0.001, PC1 explained 38.61%, PC2 explained 23.01%), and the microbial community structure in the Wec1000B group before intervention (Wec1000B_0) and after intervention (Wec1000B_1) also showed a significant separation trend in PCoA (PERMANOVA p value = 0.048, PC1 explained 16.61%, PC2 explained 27.08%), suggesting that the differences in microbial community composition were statistically significant; the sample points of the two groups showed a clear clustering in the two-dimensional sorting diagram, indicating an increase in the richness of the intestinal microbiota, an increase in the number of microbial species, and possibly an improvement in microbial community uniformity, with a more stable structure, which is conducive to maintaining the stability of the intestinal microecology.

#### 3.4.2 Alteration of microbial community structure

**Figure 5:**
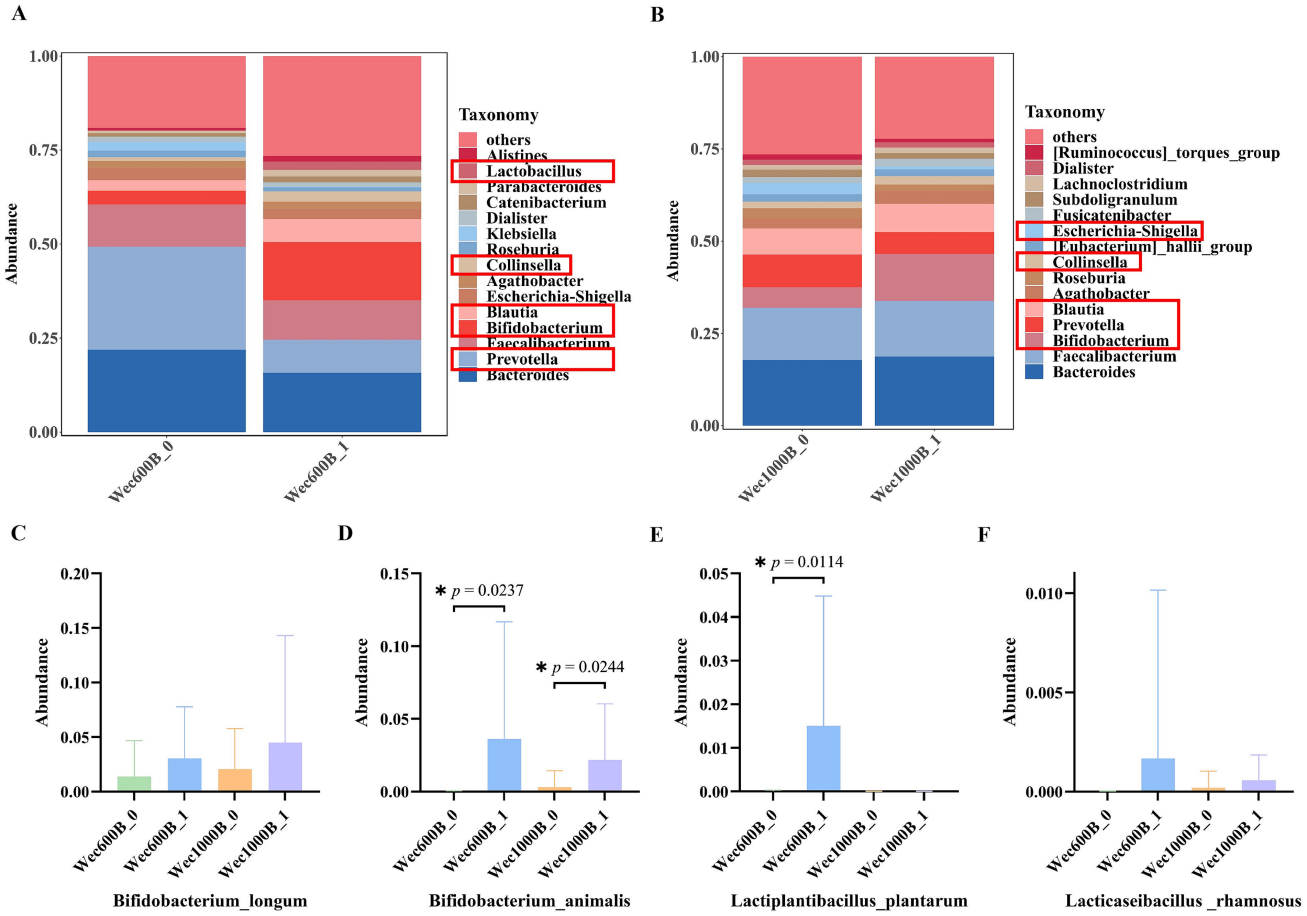
The distribution of microbial community structure and the changes in added strains at the species level after intervention. **(A)** Relative abundance of bacterial communities at the genus level in the Wec600B group. **(B)** Relative abundance of bacterial communities at the genus level in the Wec1000B group. **(C)** Add the strain *Bifidobacterium _longum*. **(D)** Add the strain *Bifidobacterium _animalis*. **(E)** Add the strain *Lactiplantibacillus _plantarum*; **(F)** Add the strain *Lacticaseibacillus _rhamnosus*. Wec600B_0 and Wec1000B_0 represent the pre-intervention status of the Wec600B group and the Wec1000B group; Wec600B_1 and Wec1000B_1 represent the post-intervention status of the Wec600B group and the Wec1000B group.

As shown in Figures 5A,B, the relative abundance of intestinal microbiota at the genus level for different groups is presented, only showing the top 15 most abundant genera. This study explores the impact of the intervention on the structure of the intestinal microbiota. The results indicate that before the intervention (Wec600B_0, Wec1000B_0), the bacterial genera such as *Bacteroides*, *Prevotella*, *Faecalibacterium*, *Bifidobacterium*, and *Blautia* occupied certain proportions in the intestinal microbiota. After the intervention (Wec600B_1 group and Wec1000B_1 group), the relative abundances of beneficial bacterial genera such as *Bifidobacterium*, *Lactobacillus*, *Blautia*, and *Collinsella* significantly increased; at the same time, the proportions of some potential harmful bacterial genera decreased, such as *Prevotella*, *Escherichia-Shigella*, and *Klebsiella*. The decrease in abundance directly reduced the risk of intestinal infection and improved symptoms of diarrhea and abdominal pain. By analyzing the top 200 bacterial strains with relatively high relative abundance at the species level, we explored the changes in the added bacterial strains before and after the intervention with high-potency multi-strain probiotic formulations. We identified two groups of added bacterial strains, including *Bifidobacterium_longum*, *Bifidobacterium_animalis*, *Lactiplantibacillus_plantarum*, *Lacticaseibacillus _rhamnosus*, as shown in Figures 5C–F. Among them, the *Bifidobacterium_animalis* in both groups and *Lactiplantibacillus_plantarum* in the Wec600B group showed significant differences in relative abundance after the intervention (*p* < 0.05). The relative abundances of the other two strains also showed a certain upward trend after the intervention. The results indicate that both groups of combined bacterial strains are rich in a high proportion of *Bifidobacterium_longum* and *Bifidobacterium_animalis*, and the relative abundances of *Lactiplantibacillus_plantarum* and *Lacticaseibacillus_rhamnosus* in the Wec600B group are higher than those in the Wec1000B group, suggesting that high-potency multi-strain probiotics can specifically regulate the abundance of certain bacterial strains and improve gastrointestinal discomfort.

**Figure 6:**
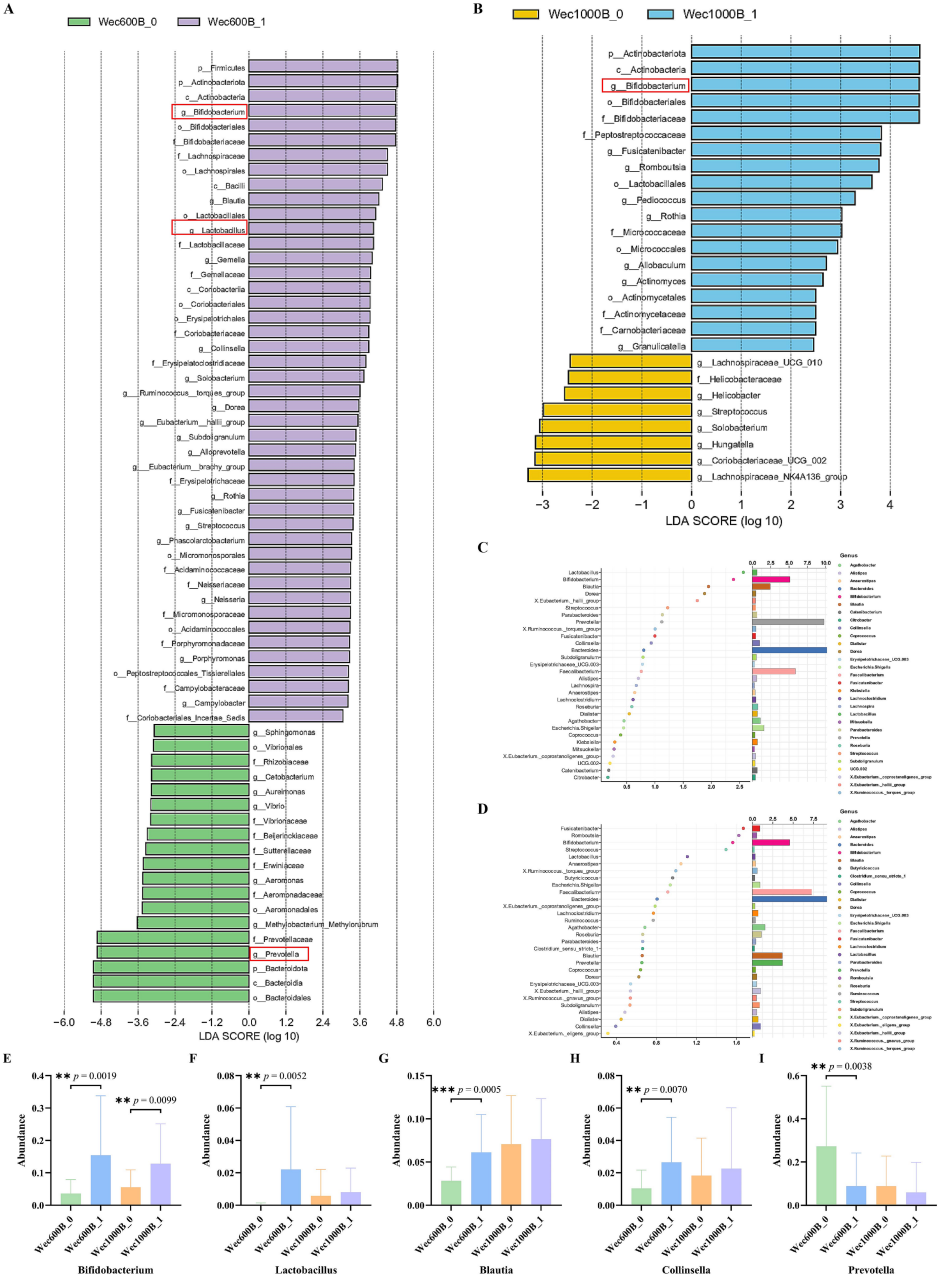
Changes in the microbiota after intervention. **(A)** LEfSe analysis of the Wec600B group. **(B)** LEfSe analysis of the Wec1000B group. **(C)** Random forest analysis of the Wec600B group. **(D)** Random forest analysis of the Wec1000B group. **(E)** Different genus *Bifidobacterium*. **(F)** Different genus *Lactobacillus*. **(G)** Different genus *Blautia*. **(H)** Different genus *Collinsella*. **(I)** Different genus *Prevotella*. Wec600B_0 and Wec1000B_0 represent the pre-intervention status of the Wec600B group and the Wec1000B group; Wec600B_1 and Wec1000B_1 represent the post-intervention status of the Wec600B group and the Wec1000B group. *p < 0.05; **p < 0.01; ***p < 0.001.

As shown in Figures 6A,B, through LEfSe analysis, the composition of species that differ between two or more communities was revealed, and the regulatory effects of the intervention on the intestinal microbiota before and after were explored. The results showed that after the intervention, in the Wec600B_1 group, the genera *Bifidobacterium* (*Bifidobacterium*) and *Lactobacillus* were significantly enriched (LDA > 3.6, *p* < 0.05), while the abundance of the *Prevotella* genus decreased; in the Wec1000B_1 group after the intervention, the abundance of *g__Bifidobacterium* (*Bifidobacterium* genus) significantly increased (LDA > 4.0, *p* < 0.05), indicating that its abundance was significantly enriched in this group, verifying the changes in the structure of the intestinal microbiota at the genus level before and after the intervention as previously analyzed. As shown in Figures 6C,D, random forest analysis is a core tool for quantifying the significance and effect size of inter-group differences, and is applicable for statistical inference of changes in microbial community abundance in microbiomics. The forest plot analysis was used again to verify the results of LEfSe analysis. It indicated that the Wec600B_1 and Wec1000B_1 combined probiotics reshaped the intestinal microecology and improved gastrointestinal function through different strain combinations. According to Figures 6E–I, through the above analysis, Two groups of prominent differential strains were identified, such as *Bifidobacterium*, *Lactobacillus*, *Blautia*, *Collinsella*, and *Prevotella*. These strains were compared before and after the intervention. It was found that after 4 weeks of intervention, *Bifidobacterium* (40), *Lactobacillus*, *Blautia*, and *Collinsella* significantly increased; while *Prevotella* significantly decreased.

**Figure 7:**
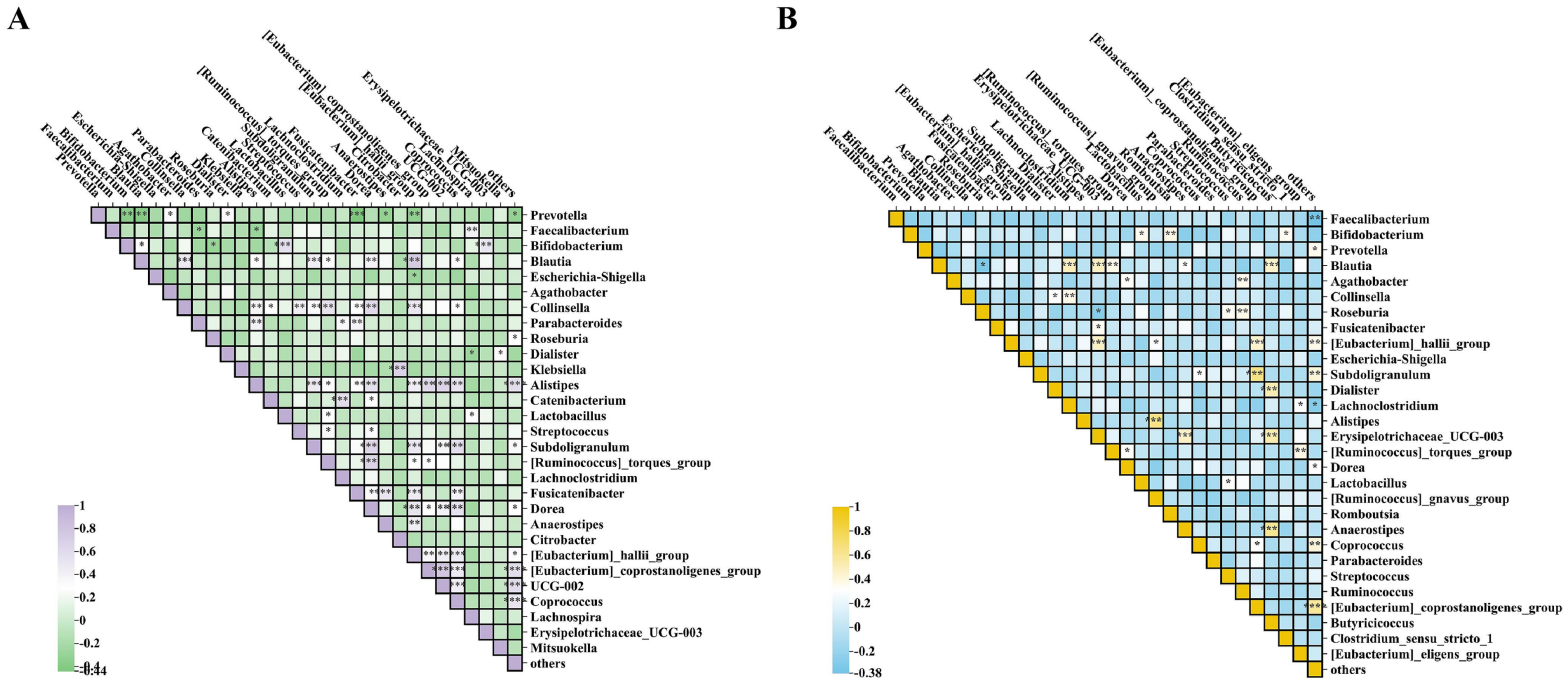
Analysis of inter-microbial correlations post-intervention. **(A)** Heatmap of the Wec600B group. **(B)** Heatmap of the Wec1000B group. *p < 0.05; **p < 0.01; ***p < 0.001.

As shown in Figures 7A,B, through heatmap analysis of the interactions among the bacterial communities after the intervention, it was found that beneficial bacteria such as *Bifidobacterium* and *Lactobacillus* showed a significant positive correlation with each other, while they had a negative correlation with harmful bacteria such as *Prevotella* and *Escherichia-Shigella*. Among them, the beneficial bacteria in the Wec600B group were significantly negatively correlated with the harmful *Prevotella* bacteria, indicating that both groups of high-potency multi-strain probiotics can increase beneficial bacteria and inhibit harmful bacteria, thereby improving gastrointestinal health.

**Figure 8:**
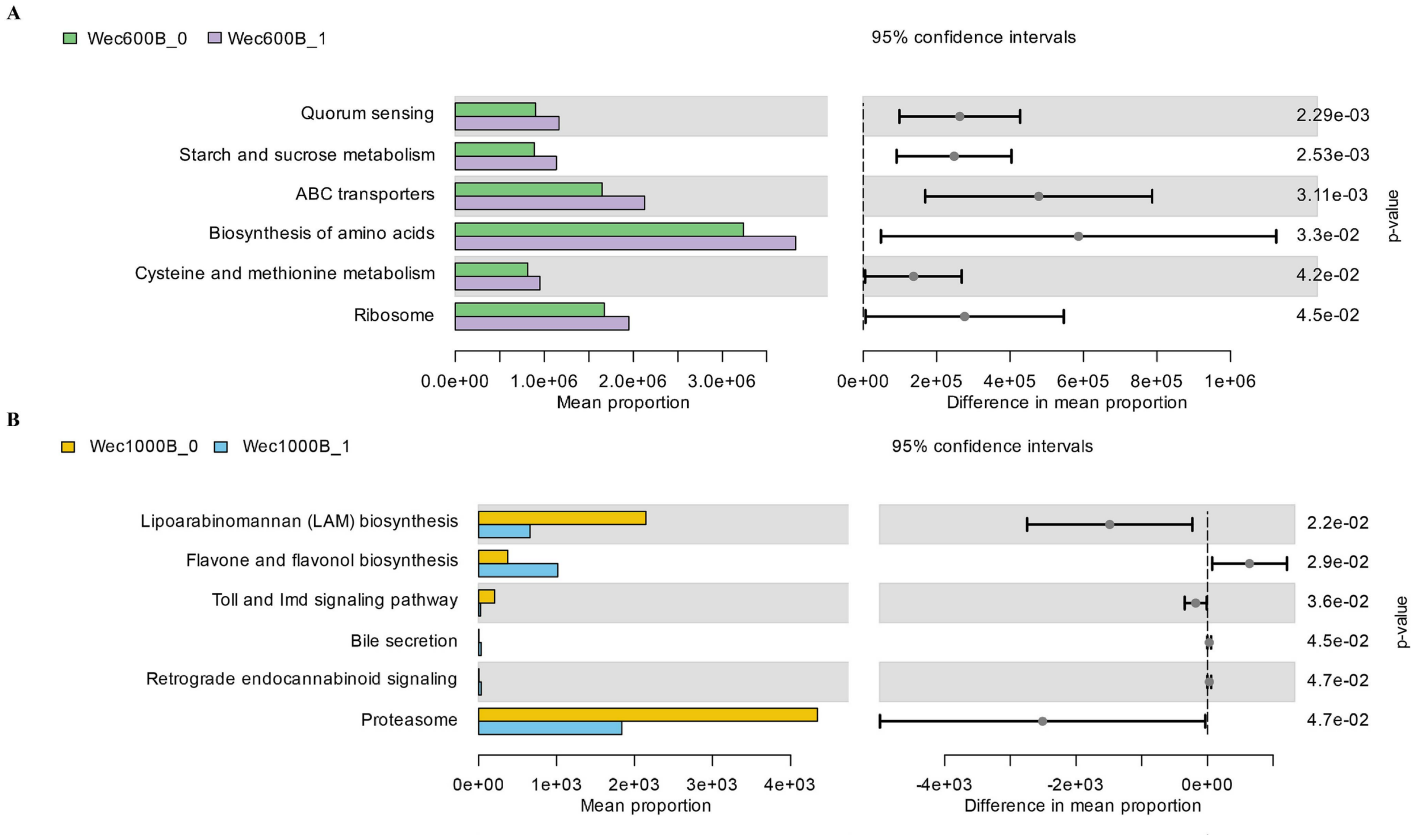
KEGG L3 metabolic pathway. **(A)** The KEGG L3 metabolic pathway of the Wec600B group. **(B)** The KEGG L3 metabolic pathway of the Wec1000B group. Wec600B_0 and Wec1000B_0 represent the pre-intervention status of the Wec600B group and the Wec1000B group; Wec600B_1 and Wec1000B_1 represent the post-intervention status of the Wec600B group and the Wec1000B group. *p < 0.05; **p < 0.01; ***p < 0.001.

The microbial community was predicted and analyzed at the KEGG L3 level through the PICRUSt function. Combined with the changes in the microbial community and indicators, the regulatory mechanism of the intervention on the intestinal microecology and functions was revealed. As shown in Figures 8A,B, at the KEGG L3 level, each group, respectively, revealed six different pathways. The Wec600B group significantly enriched Quorum sensing, Starch and sucrose metabolism, ABC transporters, Biosynthesis of amino acids, Cysteine and methionine metabolism, and Ribosome; the main differential metabolic pathways of the Wec1000B group included Lipoarabinomannan (LAM) biosynthesis, Toll and Imd signaling pathway, Proteasome, Flavone and favonol biosynthesis, Retrograde endocannabinoid signaling, and Bile secretion. Different KEGG pathways shape the microbial community structure to promote beneficial bacteria and inhibit harmful bacteria, and synergistically regulate IgA immunity enhancement, improving FL, FC, NGAL, LPS, DAO, D - LA and other inflammatory and intestinal barrier indicators, demonstrating the positive remodeling of intestinal microecology and functions after the intervention with high-potency multi-strain probiotics.

### 3.5 Correlation analysis

**Figure 9:**
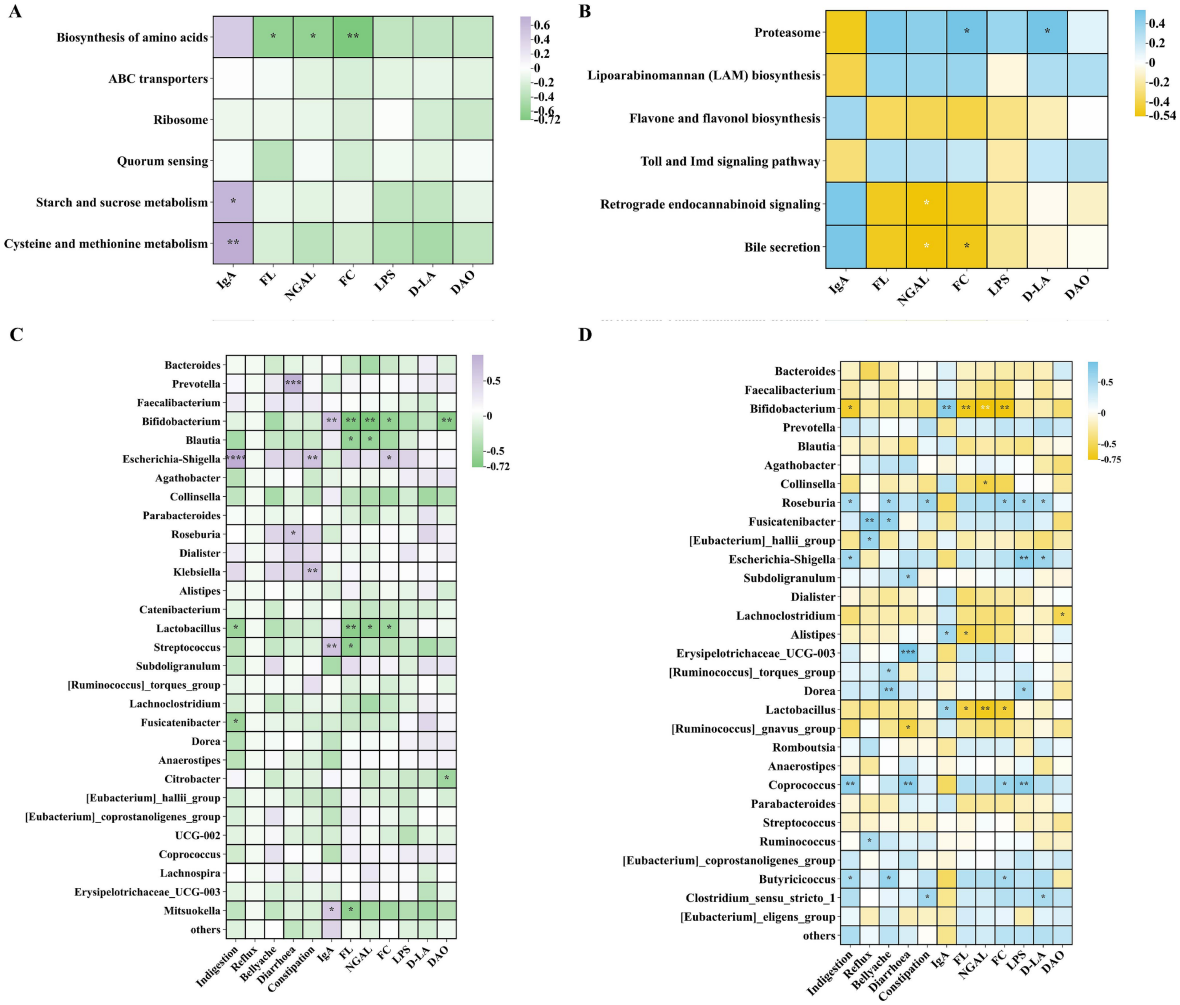
Correlation analysis. **(A)** Correlation analysis of KEGG L3 metabolic pathways in the Wec600B group and clinical indicators. **(B)** Correlation analysis of KEGG L3 metabolic pathways in the Wec1000B group and clinical indicators. **(C)** Correlation analysis of the microbiota in the Wec600B group and questionnaires as well as clinical indicators. **(D)** Correlation analysis of the microbiota in the Wec1000B group and questionnaires as well as clinical indicators. Wec600B_0 and Wec1000B_0 represent the Wec600B group and Wec1000B group before intervention; Wec600B_1 and Wec1000B_1 represent the Wec600B group and Wec1000B group after intervention. *p < 0.05; **p < 0.01; *** *p* < 0.001.

Through correlation analysis of clinical indicators with KEGG L3 metabolic pathways, microbiota with questionnaires, and clinical indicators, this study investigated the ameliorative effects of high-potency multi-strain probiotics on gastrointestinal dysfunction. As illustrated in Figures 9A,B, significant positive correlations were observed between IgA and the metabolic pathways of starch and sucrose metabolism, as well as cysteine and methionine metabolism in the Wec600B group. Conversely, FL, FC, and NGAL exhibited significant negative correlations with the biosynthesis of amino acids. In the Wec1000B group, FC and NGAL demonstrated significant negative correlations with retrograde endocannabinoid signaling and bile secretion, while proteasome showed a negative correlation with IgA and positive correlations with markers of inflammation and intestinal injury. In Figures 9C,D, it can be observed that the beneficial differential bacterial groups *Bifidobacterium*, *Lactobacillus*, *Blautia*, and *Collinsella* in both groups have a negative correlation with the questionnaire assessment indicators, a positive correlation with IgA, and a negative correlation with inflammatory indicators and intestinal barrier damage markers. The harmful differential bacterial group *Prevotella* has a positive correlation with the questionnaire assessment indicators, a negative correlation with IgA, and a positive correlation with inflammatory indicators and intestinal barrier damage markers. Especially, the beneficial differential bacterial groups *Bifidobacterium* and *Lactobacillus* in both groups have a significant correlation with clinical indicators. Through the above correlation analysis, it is found that the correlation between the Wec1000B group and clinical indicators is stronger and more significant. This indicates that the higher-potency multi-strain probiotic intervention has a strong correlation with the body’s immunity, inflammatory response, and intestinal barrier through the interaction of bacterial communities and metabolic pathways. Combined with the beneficial bacteria effect, it inhibits harmful bacteria, thereby better exerting the role of improving gastrointestinal discomfort symptoms.

## 4 Discussion

GI dysfunction affects multiple systems such as circulation and respiration. In severe cases, it disrupts the epithelial-microbial balance and causes inflammation, triggering multiple organ dysfunction. According to the investigation, about 60% of critically ill patients have GI dysfunction and are associated with adverse outcomes (41). Since the vast majority of the microbial flora exist in the gastrointestinal tract, their interaction disorder with the host can lead to various gastrointestinal diseases (42). Studies have shown that probiotic strains interact with the host through different mechanisms and can alleviate clinical symptoms, and even intervene in the pathological process of diseases under specific conditions (43). Probiotics regulate the intestinal microenvironment, increase the proportion of beneficial bacteria, inhibit harmful bacteria, and maintain barrier function (44), thereby reducing intestinal inflammation, inhibiting inflammatory factors, and regulating immune function (45). In this study, after two groups of high-potency multi-strain probiotic formulations were administered for 4 weeks, the gastrointestinal discomfort symptoms of the study participants could be significantly improved. The safety of Wec600B and Wec1000B high-potency multi-strain probiotic formulations were evaluated. After intervention with Wec600B, the scores of the study participants decreased compared to the baseline, with extremely significant reductions in symptoms of indigestion and diarrhea (*p* < 0.0001), significantly reduced reflux (*p* < 0.01) and abdominal pain symptoms (*p* < 0.05), and no significant difference in constipation (*p* > 0.05). This indicates that the Wec600B group had a significant improvement in symptoms of indigestion and diarrhea, while there was no improvement in constipation symptoms. After intervention with Wec1000B, the scores of the study participants also decreased significantly compared to the baseline, with extremely significant reductions in symptoms of indigestion and constipation (*p* < 0.0001), extremely significant reductions in abdominal pain and diarrhea symptoms (*p* < 0.001), and significantly reduced reflux symptoms (*p* < 0.01). This shows that the Wec1000B group had an extremely significant effect in improving symptoms of indigestion, diarrhea, abdominal pain and constipation, and demonstrated that probiotic intervention could improve gastrointestinal motility and defecation function. Compared to the Wec600B group, the Wec1000B group showed more significant effects in improving gastrointestinal symptoms, being more comprehensive. Next, we will discuss the immune system of individuals with gastrointestinal disorders. Research has shown that probiotic intervention can increase the level of IgA in feces, protect the intestines from harmful substances, and regulate the immune system (46, 47).

Through this study, it was found that the level of immune factor IgA significantly increased in both groups after high-potency multi-strain probiotic intervention, and FL decreased. This indicates that probiotics can, to a certain extent, enhance the body’s immunity. There are also studies indicating that the reduction of inflammatory factors such as calprotectin FC and intestinal damage markers can decrease intestinal inflammation and intestinal permeability (48), and enhance immunity (49). This study found that after 4 weeks of intervention with the high-potency multi-strain probiotic formulations, the levels of inflammatory factors such as calcium-binding protein FC and neutrophil gelatinase-associated lipocalin NGAL, as well as intestinal damage markers such as diamine oxidase (DAO), D-lactic acid (D-LA), and lipopolysaccharide (LPS) (50), could be reduced in the body. This provides new evidence for subsequent research on the ability of high-potency multi-strain probiotics to reduce inflammation and enhance intestinal barrier function, and also lays a foundation for further research on the regulation of intestinal metabolomics by high-potency multi-strain probiotics.

In order to explore the mechanism by which the high-potency Wec600B and Wec1000B multi-strain probiotic formulations improve gastrointestinal discomfort symptoms, we then analyzed the intestinal flora. Through 16S rRNA flora sequencing analysis, the two groups of intestinal flora contained certain proportions of bacterial genera such as *Bacteroides*, *Prevotella*, *Faecalibacterium*, *Bifidobacterium*, and *Blautia*.

After the intervention, the relative abundances of beneficial bacterial genera such as *Bifidobacterium*, *Lactobacillus*, *Blautia*, and *Collinsella* significantly increased in both groups. Among them, *Bifidobacterium* can increase the frequency of defecation, improve the consistency of feces, induce beneficial changes in the intestinal microbiota, inhibit the adhesion of pathogenic bacteria, and reduce intestinal infections. It has a remarkable effect on children’s intestinal health and the improvement of constipation (51). This is consistent with the significant improvement in constipation symptoms in the Wec1000B group after the intervention in the questionnaire-based gastrointestinal symptom indicators, indicating that *Bifidobacterium* is enriched after high-potency multi-strain probiotic intervention. In the correlation analysis, it inhibits harmful bacteria through positive interactions with beneficial bacteria, enhances immunity, reduces inflammation, changes the intestinal microbiota, and improves intestinal function. Some studies have also demonstrated that *Lactobacillus* produces acids to inhibit the colonization of harmful bacteria (such as *Escherichia coli*), secretes bacteriocins and organic acids, strengthens the intestinal barrier, and can regulate immunity, reduces the release of inflammatory factors (52), regulates intestinal motility, and through correlation analysis, it shows a significant negative correlation with inflammatory factors and questionnaire-based gastrointestinal symptom indicators, indicating that *Lactobacillus* inhibits harmful bacteria, reduces inflammation, and improves gastrointestinal health. Regarding the intestinal environment of ulcerative colitis, *Blautia* may regulate the balance of the intestinal microbiota, inhibit the excessive proliferation of *Escherichia-Shigella* and other microorganisms in the intestine, reduce intestinal inflammatory responses, maintain the integrity of the intestinal barrier and alleviate symptoms (53). This indicates that the increase in *Blautia* abundance after high-potency multi-strain probiotic intervention also has this improvement effect for people with gastrointestinal disorders in this study. Then, another beneficial differential strain, *Collinsella*, which can regulate microbial balance and improve the clinical outcome of tuberculosis symptoms (54), showed an increase in *Collinsella* abundance after high-potency multi-strain probiotic intervention, indicating that *Collinsella* can regulate the microbiota balance and reduce inflammatory responses. At the same time, the proportions of some potentially harmful bacterial genera decreased, such as *Prevotella* (55), *Escherichia-Shigella* (56), and *Klebsiella* (57). These harmful bacteria can cause diarrhea and intestinal infections, and the decrease in abundance directly reduces the risk of intestinal infections, improves diarrhea and abdominal pain symptoms, and improves gastrointestinal dysfunction.

At the species level, the added bacterial strains *Bifidobacterium_longum*, *Bifidobacterium_animalis*, *Lactobacillus_plantarum*, and *Lactobacillus_rhamnosus* were analyzed. Previous studies have shown that using a high-dose oral treatment of live *Bifidobacterium_longum* can promote systemic immune regulation, increase immunoglobulin levels, improve intestinal permeability disorders, and thereby alleviate clinical symptoms (58). Some research also indicates that *Lactobacillus_plantarum* has a strong ability to colonize the intestine and significant immune regulatory effects (59). It was found that both the two groups of combined bacterial strains were rich in a high proportion of *Bifidobacterium_longum* and *Bifidobacterium_animalis*. The relative abundance of *Lactiplantibacillus _plantarum* and *Lacticaseibacillus _rhamnosus* in the Wec600B group was higher than that in the Wec1000B group. This conclusion indicates that the high-potency multi-strain probiotics contain a large number of live bacterial strains, which can enhance their colonization ability in the human intestinal tract (60), regulate the ecological balance of the gastrointestinal flora (61), and exhibit the characteristics of high-potency multi-strain probiotics.

Then, based on the functional prediction analysis of various microbial communities using PICRUSt, it was found that in the KEGG L3 level pathways, there are pathways associated with the intestinal microecology. The six metabolic pathways in the Wec600B group were significantly enriched, related to Biosynthesis of amino acids, Ribosome, ABC transporters, etc. By improving the intestinal barrier, regulating the immune system, and reducing inflammation, it has a repairing effect on intestinal health. In the KEGG L3 level analysis of the Wec1000B group, Retrograde endocannabinoid signaling reshaped the intestinal microbiota and activated the CB2 receptor pathway to alleviate inflammation and repair the intestinal barrier (62). The LAM biosynthesis pathway (63) was found to inhibit the body’s immunity, induce the production of inflammatory cytokines, and trigger inflammatory responses. However, in this study, it was found that after probiotic intervention, the enrichment decreased, which could improve immunity and reduce inflammatory responses, and had the same anti-inflammatory and immune regulation effects as the Flavone and favonol biosynthesis (64) intervention. In addition, the Proteasome pathway can activate the inflammatory pathway, inhibit autophagy, and exacerbate chronic low-grade inflammation, affecting the body’s metabolic homeostasis (65). In the correlation analysis, it was found that the microbiota was related to the questionnaires and clinical indicators. The different strains played an important role in improving intestinal health. Especially *Bifidobacterium* (*Bifidobacterium_longum*, *Bifidobacterium_animalis*) and *Lactobacillus* (*Lactobacillus_plantarum*, *Lactobacillus_rhamnosus*) showed a significant increase in abundance after intervention. These were the bacterial strains added with high-potency multi-strain probiotics, which were enriched in the intestinal tract, increasing the activity of the microbiota and forming a positive interaction with beneficial bacteria, inhibiting harmful bacteria, to reduce gastrointestinal symptoms, improve immunity, reduce inflammation, and improve the clinical outcomes of patients with gastrointestinal symptoms.

Through the comprehensive analysis of the previous two sets of data, it was found that the high-potency multi-strain probiotics in the Wec1000B group showed a more significant overall improvement effect. This indicates that the more active probiotic strains can more effectively enhance immunity, reduce inflammatory responses, repair intestinal damage, and have a more prominent effect on improving gastrointestinal dysfunction. In conclusion, high-potency multi-strain probiotic formulations can regulate human intestinal homeostasis through multiple pathways, promote intestinal health, and thereby improve gastrointestinal function. This study confirms the potential of high-potency multi-strain probiotic formulations in ameliorating abnormal gastrointestinal function, making a certain contribution to the research field in this area and providing a new perspective or approach for clinical application. However, this study has certain limitations. For instance, although many other indicators reflecting gastrointestinal function require blood sample testing, only fecal samples were selected for analysis in this study. Additionally, the current study only analyzed the intestinal flora. Future research could focus on integrating metabolomics technology to further explore the probiotic-host interaction mechanisms, systematically analyze the dynamic changes of short-chain fatty acids, bile acids, and microbial metabolites, and clarify the molecular pathways through which probiotics affect nutrient absorption, immune regulation, and energy metabolism via the “microbiota-gut-metabolism axis.” Furthermore, by combining metagenomics/transcriptomics to construct a multi-omics network, the synergistic mechanisms of bacterial strains and their remodeling effects on intestinal barrier function could be deciphered.

## 5 Conclusion

This study conducted an in-depth investigation into the improvement effects and safety evaluation of the high- potency Wec600B and Wec1000B probiotic formulations on 100 individuals with gastrointestinal dysfunction. The results showed that both probiotics could significantly reduce the levels of immune-related factors (FL), inflammatory factors (NGAL, FC), and intestinal damage markers (LPS, D-AL, DAO) in the feces, and increase the content of immune factor IgA. The mechanism by which the Wec600B and Wec1000B high-potency multi-strain probiotic formulations intervention improves gastrointestinal dysfunction may be due to the influence of key strains (*Bifidobacterium*, *Lactobacillus*, *Blautia*, *Collinsella*, and *Prevotella*). The safety evaluation indicates that the probiotic preparation shows good tolerance in improving gastrointestinal discomfort symptoms, without any serious adverse reactions, and can effectively relieve abdominal pain, abdominal distension, etc., and improve constipation and diarrhea conditions. These results can serve as scientific evidence to prove that the Wec600B and Wec1000B high-potency multi-strain probiotic formulations intervention can alleviate gastrointestinal discomfort symptoms. Future research may focus on exploring other aspects of the Wec1000B high-potency multi-strain probiotic formulations, and also conduct further studies on the long-term intervention of probiotics, evaluate the safety assessment and improvement effects of the high-potency multi-strain probiotics.

## Data Availability

The datasets presented in this study can be found in online repositories. The names of the repository/repositories and accession number(s) can be found at: https://www.ncbi.nlm.nih.gov/, bioproject/PRJNA1312311.
